# Pharmacological Inhibition of Caspase-1 Ameliorates Cisplatin-Induced Nephrotoxicity through Suppression of Apoptosis, Oxidative Stress, and Inflammation in Mice

**DOI:** 10.1155/2018/6571676

**Published:** 2018-12-23

**Authors:** Jung-Yeon Kim, Jae-Hyung Park, Kiryeong Kim, Jungmin Jo, Jaechan Leem, Kwan-Kyu Park

**Affiliations:** ^1^Department of Immunology, School of Medicine, Catholic University of Daegu, Daegu 42472, Republic of Korea; ^2^Department of Physiology, School of Medicine, Keimyung University, Daegu 42601, Republic of Korea; ^3^Department of Hematology-Oncology, Inje University Seoul Paik Hospital, Seoul 04551, Republic of Korea; ^4^Department of Pathology, School of Medicine, Catholic University of Daegu, Daegu 42472, Republic of Korea

## Abstract

Caspase-1 is a proinflammatory caspase responsible for the proteolytic conversion of the precursor forms of interleukin-1*β* to its active form and plays an important role in the pathogenesis of various inflammatory diseases. It was reported that genetic deficiency of caspase-1 prevented cisplatin-induced nephrotoxicity. However, whether pharmacological inhibition of caspase-1 also has a preventive effect against cisplatin-induced kidney injury has not been evaluated. In this study, we examined the effect of Ac-YVAD-cmk, a potent caspase-1-specific inhibitor, on renal function and histology in cisplatin-treated mice and explored its underlying mechanisms. We found that administration of Ac-YVAD-cmk effectively attenuated cisplatin-induced renal dysfunction, as evidenced by reduced plasma levels of blood urea nitrogen and creatinine, and histological abnormalities, such as tubular cell death, dilatation, and cast formation. Administration of Ac-YVAD-cmk inhibited caspase-3 activation as well as caspase-1 activation and attenuated apoptotic cell death, as assessed by terminal deoxynucleotidyl transferase-mediated dUTP nick-end labeling, in the kidneys of cisplatin-treated mice. Cisplatin-induced G2/M arrest of renal tubular cells was also reduced by caspase-1 inhibition. In addition, administration of Ac-YVAD-cmk reversed increased oxidative stress and depleted antioxidant capacity after cisplatin treatment. Moreover, increased macrophage accumulation and elevated expression of cytokines and chemokines were attenuated by caspase-1 inhibition. Taken together, these results suggest that caspase-1 inhibition by Ac-YVAD-cmk protects against cisplatin-induced nephrotoxicity through inhibition of renal tubular cell apoptosis, oxidative stress, and inflammatory responses. Our findings support the idea that caspase-1 may be a promising pharmacological target for the prevention of cisplatin-induced kidney injury.

## 1. Introduction

Cisplatin is one of the most important chemotherapeutic agents widely used in the treatment of many human cancers [1, 2]. However, serious side effects in normal tissues, particularly nephrotoxicity in the kidneys, limit the use of cisplatin. Although some strategies including hydration management are used to prevent cisplatin-induced nephrotoxicity, there is currently no specific treatment available. Over the last few decades, much effort has been made to reveal the pathogenesis of cisplatin-induced nephrotoxicity, because understanding its underlying mechanisms will guide the development of novel therapeutic strategies. It has been suggested that renal tubular cell apoptosis, oxidative stress, and inflammatory responses are critical factors that contribute to the development of cisplatin-induced nephrotoxicity, although the exact mechanisms remain incompletely understood [1, 2].

Caspases are a family of cysteine proteases that play central roles in the maintenance of cellular and organismal homeostasis by acting as key mediators of apoptosis and inflammatory response [3]. Among them, caspase-1 is a proinflammatory caspase responsible for the proteolytic conversion of the precursor forms of interleukin- (IL-) 1*β* to its active form [4]. IL-1*β*, activated by caspase-1, induces inflammatory responses [5] and is involved in the generation of oxidative stress [6]. Caspase-1 also can execute cell death processes such as pyroptosis and apoptosis [4, 7]. Thus, caspase-1 may be a potential therapeutic target for various inflammatory diseases. A previous study showed that caspase-1 activity was increased during the development of cisplatin-induced kidney injury and caspase-1-deficient mice were protected from cisplatin-induced nephrotoxicity, suggesting that caspase-1 is a key mediator of cisplatin-induced kidney injury [8]. However, whether pharmacological inhibition of caspase-1 also has a preventive effect against cisplatin-induced nephrotoxicity has not been evaluated.

In the present study, we found that administration of Ac-YYAD-cmk, a potent caspase-1-specific inhibitor, prevented cisplatin-induced kidney injury in mice. This effect was associated with reduced apoptosis, oxidative stress, and inflammatory responses in the kidneys. These results suggest that caspase-1 may be a promising target for the prevention of cisplatin-induced nephrotoxicity.

## 2. Materials and Methods

### 2.1. Animal Experiments

Eight-week-old male C57BL/6 mice were purchased from Samtako (Daejeon, South Korea) and randomly divided into three groups, as follows: control (Con, *n* = 8), cisplatin alone (CP, *n* = 8), and cisplatin plus AC-YYAD-cmk (CP + YVAD, *n* = 8). For cisplatin treatment, mice were given a single intraperitoneal injection of cisplatin (Sigma-Aldrich, St. Louis, MO, USA; dissolved in 0.9% normal saline) at a dose of 15 mg/kg. To evaluate the effects of AC-YYAD-cmk (Cayman Chemical, Ann Arbor, MI, USA) on cisplatin-induced nephrotoxicity, mice were injected intraperitoneally with 10 mg/kg AC-YYAD-cmk for 3 days, starting 1 h prior to a single dose of cisplatin. The dose of AC-YYAD-cmk was determined based on the results of a previous study [9]. Mice were sacrificed 3 days after cisplatin injection, and blood and kidney tissue samples were collected. Mice were housed at ambient temperature (20–22°C) under a 12 h : 12 h light-dark cycle with free access to water and food. Animal care and all experimental procedures were approved and conducted in accordance with the guidelines of the Institutional Animal Care and Use Committee of the Catholic University of Daegu.

### 2.2. Plasma and Tissue Biochemical Assays

Plasma levels of blood urea nitrogen (BUN) and creatinine were measured using a BUN assay kit (Asan Pharmaceutical, Seoul, South Korea) and the QuantiChrom Creatinine Assay Kit (Bioassay Systems, Hayward, CA, USA), respectively, according to the manufacturer's instructions. Levels of malondialdehyde (MDA) were measured using the Lipid Peroxidation (MDA) Assay Kit (Sigma-Aldrich), and the ratio of reduced to oxidized glutathione (GSH/GSSG) was assessed using the Glutathione Detection Kit (Enzo Life Sciences, Farmingdale, NY, USA), both according to the manufacturer's instructions.

### 2.3. Histological and Immunohistochemical Staining

The kidneys were rapidly removed from each mouse. The tissues were immediately fixed in 4% paraformaldehyde and embedded in paraffin. Serial sections were deparaffinized in xylene, rehydrated using descending grades of ethanol, and stained with hematoxylin and eosin (H&E) and periodic acid-Schiff (PAS). Images were captured using the NIKON A1+ confocal microscope (Nikon, Tokyo, Japan). Tubular damage in PAS-stained kidney sections was scored at a ×200 magnification using 10 randomly selected fields for each kidney according to the level of cortical tubular injury, as previously described: 0, normal; 1, 1–10%; 2, 11–25%; 3, 26–45%; 4, 46–75%; and 5, 76–100% [10].

For immunohistochemistry, the sections were incubated with primary antibodies (Abcam, Cambridge, MA, USA) against 4-hydroxynonenal (4-HNE) or Mac-2 overnight at 4°C and were then incubated with a secondary antibody for 30 min. The percentage of positive staining per field was determined using i-Solution Lite V.9.1 Image Analysis Software (IMTechnology, Vancouver, BC, Canada).

### 2.4. Terminal Deoxynucleotidyl Transferase-Mediated dUTP Nick-End Labeling (TUNEL) Assay

Apoptotic cell death was examined in the kidney sections using the in situ Cell Death Detection Kit (Roche Diagnostics, Indianapolis, IN, USA), according to the manufacturer's instructions. Briefly, the kidney sections were deparaffinized in xylene, rehydrated using descending grades of ethanol, and permeabilized for 30 min at room temperature with proteinase K in 10 mM Tris-HCl, pH 7.4–8. After washing with phosphate-buffered saline, kidney sections were incubated in the TUNEL reaction mixture for 1 h at 37°C. Nuclei were counterstained with DAPI. Images were captured using the NIKON A1+ confocal microscope. The number of TUNEL-positive cells was counted in 5 random fields for each kidney.

### 2.5. Immunofluorescence Staining

Paraffin-embedded mouse kidney sections were prepared using a routine procedure. After blocking with 10% donkey serum for 30 min, the slides were immunostained with primary antibodies against Ki67 (Millipore, Billerica, MA, USA) and phosphohistone H3 (p-H3; Cell Signaling, Danvers, MA, USA). After washing, they were incubated with secondary antibodies for 30 min at 37°C. Nuclei were counterstained with DAPI. Stained slides were imaged using the NIKON A1+ confocal microscope.

### 2.6. Western Blot Analysis

Kidney tissues were prepared using the CelLytic MT Cell Lysis Reagent (Sigma-Aldrich). Proteins were resolved by sodium dodecyl sulfate-polyacrylamide gel electrophoresis and then transferred to nitrocellulose membrane. After blocking, the membrane was incubated with the following primary antibodies: anti-cleaved caspase-1 (Cell Signaling), anti-cleaved caspase-3 (Cell Signaling), anti-tumor necrosis factor-*α* (TNF-*α*; Abcam), and anti-glyceraldehyde-3-phosphate dehydrogenase (GAPDH; Cell Signaling). The membrane was washed and incubated with horseradish peroxidase-conjugated secondary antibodies, and signals were detected using an enhanced chemiluminescence detection system (Thermo Fisher Scientific, Waltham, MA, USA). Signal intensities were measured with an image analyzer (ChemiDoc™ XRS+; Bio-Rad Laboratories, Hercules, CA, USA). GAPDH was used as a protein loading control. Relative protein expression was quantified using NIH ImageJ software.

### 2.7. Quantitative Real-Time RT-PCR

Total RNA was isolated from tissues using the TRIzol Reagent (Thermo Fisher Scientific, Waltham, MA, USA) and reverse transcribed to make cDNA by using oligo (dT) 18 primers and the AccuPower RT Premix (Bioneer, Daejeon, South Korea) according to the manufacturer's instructions. Quantitative real-time PCR was performed using the Real-Time PCR 7500 system (Applied Biosystems, Foster City, CA, USA) and Power SYBR Green PCR Master Mix (Applied Biosystems). All primer sequences are shown in Table 1. The mRNA levels of specific genes were normalized to those of GAPDH.

### 2.8. Statistical Analysis

Data are expressed as the mean ± standard error of the mean (SEM). The differences between groups were analyzed using one-way analysis of variance (ANOVA) followed by Bonferroni's post hoc test. A *P* value < 0.05 was considered statistically significant.

## 3. Results

### 3.1. Ac-YVAD-cmk Attenuated Renal Dysfunction and Tubular Injury in Cisplatin-Treated Mice

**Figure 1:**
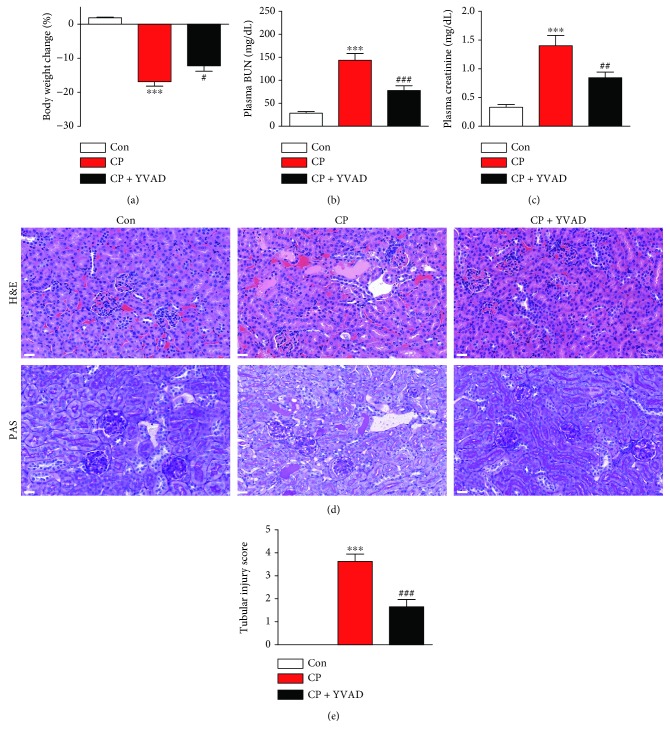
Effects of Ac-YVAD-cmk on body weight, renal function, and renal histology in cisplatin-treated mice. Mice were injected intraperitoneally with 10 mg/kg AC-YYAD-cmk for 3 days, starting 1 h prior to a single dose of cisplatin (15 mg/kg). (a) Percent change in body weight. (b) Plasma blood urea nitrogen (BUN). (c) Plasma creatinine. (d) Representative images of hematoxylin and eosin (H&E) and periodic acid-Schiff (PAS) staining on kidney sections. Scale bar: 25 *μ*m. (e) Tubular injury score. Con: control, CP: cisplatin, and CP + YVAD: cisplatin plus Ac-YVAD-cmk. *n* = 8 per group. All data are expressed as the mean ± SEM. ^∗∗∗^*P* < 0.001 vs. Con. ^#^*P* < 0.05, ^##^*P* < 0.01, and ^###^*P* < 0.001 vs. CP.

For the induction of cisplatin-induced acute kidney injury, mice were given a single intraperitoneal injection of cisplatin at a dose of 15 mg/kg. Cisplatin-treated mice exhibited reduced body weight (Figure 1(a)) and a marked deterioration of renal function, as evidenced by elevated plasma levels of BUN (Figure 1(b)) and creatinine (Figure 1(c)), compared to vehicle-treated mice. Administration of Ac-YVAD-cmk significantly attenuated the cisplatin-induced elevation of plasma BUN and creatinine levels. Histological examination revealed that cisplatin-treated mice displayed severe renal pathological changes characterized by tubular cell death, tubular dilatation, and tubular cast formation (Figures 1(d) and 1(e)). These histological abnormalities were also significantly ameliorated by administration of Ac-YVAD-cmk.

### 3.2. Ac-YVAD-cmk Prevented Apoptotic Cell Death and G2/M Cell Cycle Arrest in the Kidneys of Cisplatin-Treated Mice

**Figure 2:**
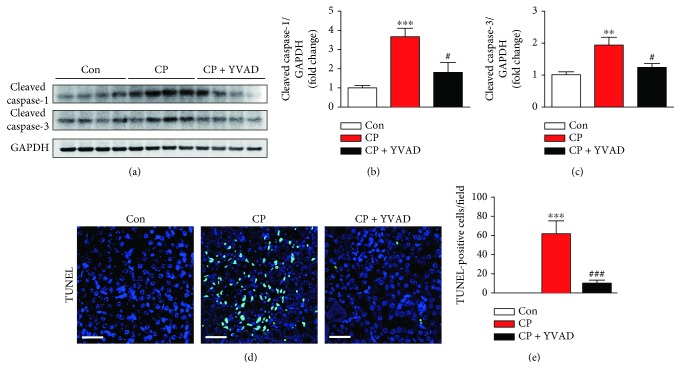
Effects of Ac-YVAD-cmk on cisplatin-induced apoptotic cell death in the kidneys. (a) Western blot analysis of the expression of cleaved caspase-1 and cleaved caspase-3 in the kidneys. Graphs show the results of quantitative analysis of cleaved caspase-1 (b) and cleaved caspase-3 (c). (d) Representative images of TUNEL staining on kidney sections. Nuclei were counterstained with DAPI. Scale bar: 50 *μ*m. (e) The number of TUNEL-positive cells in 5 random fields for each kidney. (f) Representative immunofluorescence staining of Ki67 (green) and p-H3 (red) on kidney sections. Nuclei were counterstained with DAPI. Scale bar: 50 *μ*m. (g) Percentage of cells in the G2/M phase among all proliferative (Ki67-positive) tubular epithelial cells. Con: control, CP: cisplatin, and CP + YVAD: cisplatin plus Ac-YVAD-cmk. *n* = 8 per group. All data are expressed as the mean ± SEM. ^∗∗^*P* < 0.01 and ^∗∗∗^*P* < 0.001 vs. Con. ^#^*P* < 0.05 and ^###^*P* < 0.001 vs. CP.

**Figure 3:**
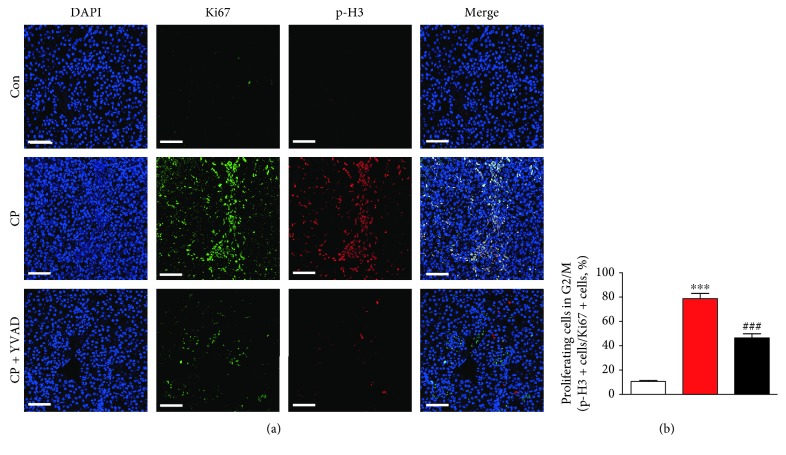
Effects of Ac-YVAD-cmk on cisplatin-induced G2/M cell cycle arrest in the kidneys. (a) Representative immunofluorescence staining of Ki67 (green) and p-H3 (red) on kidney sections. Nuclei were counterstained with DAPI. Scale bar: 50 *μ*m. (b) Percentage of cells in the G2/M phase among all proliferative (Ki67-positive) tubular epithelial cells. Con: control, CP: cisplatin, and CP + YVAD: cisplatin plus Ac-YVAD-cmk. *n* = 8 per group. All data are expressed as the mean ± SEM. ^∗∗^*P* < 0.01 and ^∗∗∗^*P* < 0.001 vs. Con. ^#^*P* < 0.05 and ^###^*P* < 0.001 vs. CP.

Apoptotic death of renal tubular cells plays a critical role in the pathogenesis of cisplatin-induced kidney injury [1, 2]. As shown in Figures 2(a)–2(c), cisplatin-treated mice exhibited increased expression of cleaved caspase-1 and caspase-3 in the kidneys, indicating that cisplatin induced the activation of both caspases. These changes were significantly suppressed by administration of Ac-YVAD-cmk. TUNEL staining also showed that the administration of Ac-YVAD-cmk significantly reduced the number of TUNEL-positive tubular cells in the kidneys of cisplatin-treated mice (Figures 2(d) and 2(e)). Moreover, treatment with cisplatin significantly increased the percentage of cells in the G2/M phase among all proliferative tubular epithelial cells, indicating that cisplatin induces G2/M arrest in the kidneys (Figures 3(a) and 3(b)). Administration of Ac-YVAD-cmk significantly reduced the increase in tubular cell G2/M arrest in the kidneys of cisplatin-treated mice.

### 3.3. Ac-YVAD-cmk Reduced Cisplatin-Induced Oxidative Stress in the Kidneys

**Figure 4:**
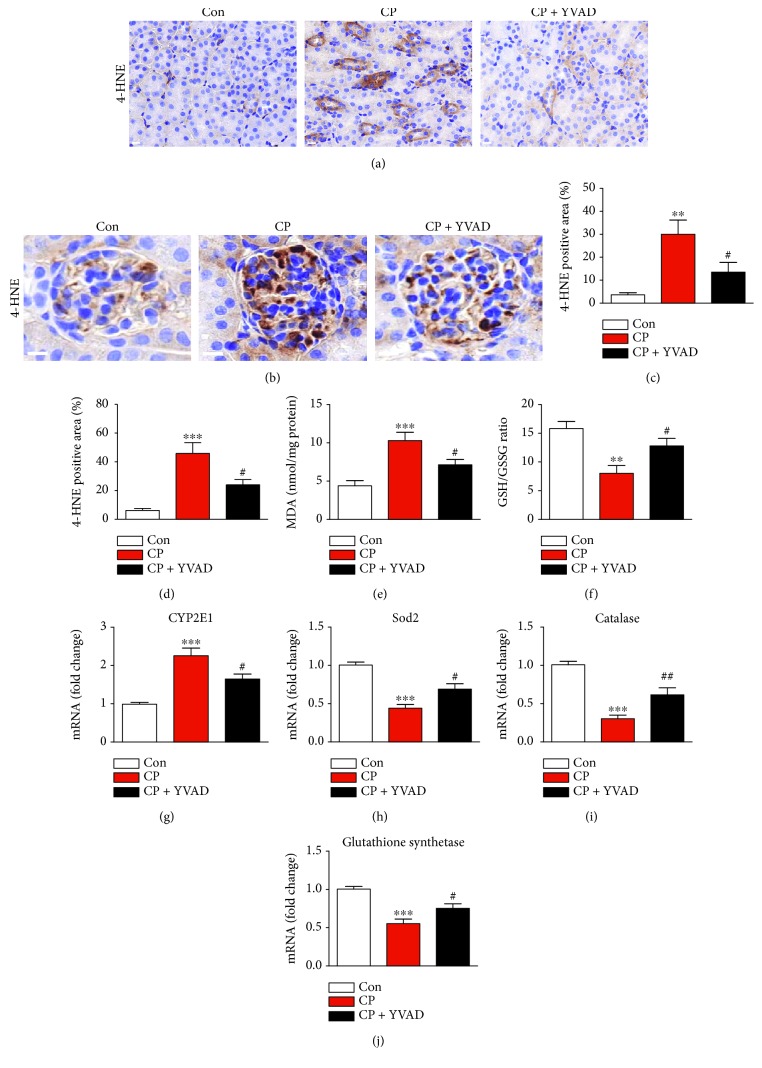
Effects of Ac-YVAD-cmk on cisplatin-induced oxidative damage in the kidneys. Representative images of immunohistochemical staining using anti-4-HNE antibody in the cortex (a) and glomerulus (b). Scale bar: 25 *μ*m. Graphs show the percentage of 4-HNE-stained area per field in the cortex (c) and glomerulus (d). (e) Level of malondialdehyde (MDA). (f) GSH/GSSG ratio. Real-time RT-PCR analysis of CYP2E1 (g), SOD2 (h), catalase (i), and glutathione synthetase (j) in the kidneys. Con: control, CP: cisplatin, and CP + YVAD: cisplatin plus Ac-YVAD-cmk. *n* = 8 per group. All data are expressed as the mean ± SEM. ^∗∗^*P* < 0.01 and ^∗∗∗^*P* < 0.001 vs. Con. ^#^*P* < 0.05 and ^##^*P* < 0.01 vs. CP.

Oxidative stress has also been implicated in the direct cellular toxicity of cisplatin [1, 2]. Immunohistochemical staining using a monoclonal antibody against 4-HNE, a marker of lipid peroxidation, showed that administration of Ac-YVAD-cmk significantly decreased the 4-HNE-positive area in both the cortex (Figures 4(a) and 4(c)) and the glomerulus (Figures 4(b) and 4(d)). The level of MDA, another marker of lipid peroxidation, in the kidneys was also significantly reduced by administration of Ac-YVAD-cmk (Figure 4(e)). In addition, the GSH/GSSG ratio, an indicator of oxidative stress, was largely decreased in the kidneys of cisplatin-treated mice compared to vehicle-treated mice and this change was significantly attenuated by administration of Ac-YVAD-cmk (Figure 4(f)). Moreover, the elevated mRNA level of cytochrome P450 2E1 (CYP2E1) (Figure 4(g)), a major prooxidant enzyme, and reduced mRNA levels of antioxidant enzymes including superoxide dismutase 2 (SOD2) (Figure 4(h)), catalase (Figure 4(i)), and glutathione synthetase (Figure 4(j)) in the kidneys of cisplatin-treated mice were significantly reversed by administration of Ac-YVAD-cmk.

### 3.4. Ac-YVAD-cmk Inhibited Cisplatin-Induced Inflammatory Responses in the Kidneys

**Figure 5:**
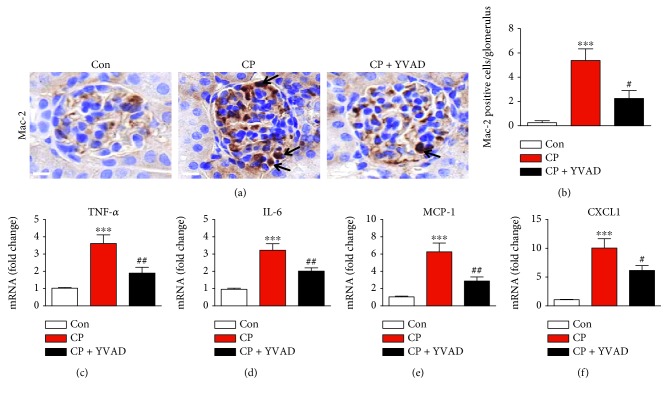
Effects of Ac-YVAD-cmk on cisplatin-induced inflammatory responses. (a) Representative images of immunohistochemical staining using anti-Mac-2 antibody in the glomerulus. Black arrows indicate Mac-2 positive cells. Scale bar: 25 *μ*m. (b) Percentage of Mac-2 positive cells per glomerulus. Real-time RT-PCR analysis of TNF-*α* (c), IL-6 (d), MCP-1 (e), and CXCL1 (f) in the kidneys. Con: control, CP: cisplatin, and CP + YVAD: cisplatin plus Ac-YVAD-cmk. *n* = 8 per group. All data are expressed as the mean ± SEM. ^∗∗∗^*P* < 0.001 vs. Con. ^#^*P* < 0.05 and ^##^*P* < 0.01 vs. CP.

Besides direct cellular toxicity, inflammation is another important pathogenic factor in cisplatin-induced nephrotoxicity [1, 2]. To examine the effect of Ac-YVAD-cmk on macrophage accumulation in the kidneys after cisplatin treatment, the kidney tissues were stained with the anti-Mac-2 antibody to identify macrophages. As shown in Figures 5(a) and 5(b), macrophage accumulation was markedly increased in the glomeruli of cisplatin-treated mice. Administration of Ac-YVAD-cmk significantly suppressed the macrophage accumulation. In addition, elevated mRNA levels of cytokines and chemokines including TNF-*α* (Figure 5(c)), IL-6 (Figure 5(d)), monocyte chemoattractant protein-1 (MCP-1) (Figure 5(e)), and chemokine (C-X-C motif) ligand 1 (CXCL1) (Figure 5(f)) in the kidneys of cisplatin-treated mice were significantly reduced by administration of Ac-YVAD-cmk.

## 4. Discussion

In the present study, we investigated the effect of Ac-YVAD-cmk, a potent caspase-1-specific inhibitor, on cisplatin-induced nephrotoxicity. We showed that administration of Ac-YVAD-cmk significantly ameliorated renal dysfunction and structural damage in the kidneys of cisplatin-treated mice. These beneficial effects of caspase-1 inhibition were associated with suppression of apoptotic cell death and G2/M arrest of tubular epithelial cells, oxidative stress, and inflammatory responses in the kidneys.

Accumulating evidence suggests that caspase-1 plays an important role in the pathogenesis of various inflammatory diseases because the protease activates proinflammatory cytokines such as IL-1*β* [4]. In this study, we observed a marked activation of caspase-1 in the kidneys after cisplatin treatment. We also found that inhibition of caspase-1 by Ac-YVAD-cmk effectively ameliorated cisplatin-induced renal dysfunction and tubular injury. Our findings are consistent with Faubel et al.'s study [8] showing that caspase-1 activity, measured by an enzymatic assay, and IL-1*β* level in the kidneys were largely increased in cisplatin-induced nephrotoxicity. They also showed that caspase-1-deficient mice were protected from cisplatin-induced kidney injury. In addition, caspase-1-deficient mice exhibited less severe kidney damage after ischemic insults [11]. Pharmacological inhibition of caspase-1 also effectively improved renal function in rats with severe acute pancreatitis [12]. Collectively, these results suggest that caspase-1 is a critical pathogenic factor leading to the development of acute kidney injury and its pharmacological inhibition has a preventive effect against cisplatin-induced nephrotoxicity.

Although the mechanisms of cisplatin-induced nephrotoxicity still remain incompletely understood, apoptosis of renal tubular cells is considered a central pathogenic process [1, 2]. Cisplatin treatment leads to the activation of Bax, which induces porous defects in the outer membrane of the mitochondria, resulting in the release of mitochondrial cytochrome c into the cytoplasm. Upon entering the cytoplasm, cytochrome c promotes the assembly of a multiprotein complex that induces proteolytic processing and activation of executioner caspases such as caspase-3. In this study, we found that Ac-YVAD-cmk significantly suppressed caspase-3 activation as well as caspase-1 activation after cisplatin treatment. The number of apoptotic tubular cells detected by TUNEL staining was markedly reduced by Ac-YVAD-cmk, suggesting the protective effect of caspase-1 inhibition against apoptotic cell death of renal tubular cells in cisplatin-induced kidney injury. These findings were in agreement with previous studies showing that caspase-1 can execute apoptosis through the activation of caspase-3 [7, 13]. In this study, we also found that cisplatin treatment increased the percentage of cells in the G2/M phase among all proliferative tubular epithelial cells, suggesting that cisplatin induces G2/M arrest in the kidneys. Previous studies showed that tubular cell G2/M arrest after acute kidney injury leads to the amplification of profibrogenic responses [14, 15]. Reversal of the G2/M arrest prevented the progression of acute kidney injury to chronic progressive fibrotic kidney disease. In this study, we observed a marked reversal of cisplatin-induced G2/M arrest by caspase-1 inhibition. These results suggest that caspase-1 inhibition by Ac-YVAD-cmk may also have a preventive effect against the development of fibrosis after acute kidney injury. Future studies will be required to examine the effects of caspase-1 inhibition on chronic kidney diseases.

Oxidative stress has also been implicated in the pathogenesis of cisplatin-induced nephrotoxicity [1, 2]. In this study, we showed that cisplatin-induced lipid peroxidation, evidenced by an increase in both the expression of 4-HNE and the amount of MDA, was markedly attenuated by Ac-YVAD-cmk. Ac-YVAD-cmk also significantly reversed cisplatin-induced reduction in the GSH/GSSG ratio. In addition, elevated mRNA expression of CYP2E1 after cisplatin treatment was significantly reduced by Ac-YVAD-cmk. Because CYP2E1 is a heme-containing enzyme that contributes to the production of reactive oxygen species (ROS), the downregulation of CYP2E1 by Ac-YVAD-cmk is possibly involved in the reduction of ROS generation and subsequent oxidative damage. In addition to ROS production, dysregulation of antioxidant systems is another important mechanism that contributes to cisplatin-induced oxidative damage [16, 17]. In this study, we also found that reduced expression of antioxidant enzymes after cisplatin treatment was significantly reversed by Ac-YVAD-cmk. Taken together, these results suggest that reduced production of ROS and increased expression of antioxidant enzymes by Ac-YVAD-cmk are involved in its protective effect against cisplatin-induced oxidative damage and kidney injury.

In addition to direct cellular toxicity, inflammatory responses contribute to the development of cisplatin-induced kidney injury [1, 2]. Particularly, macrophage infiltration into damaged kidney tissue has been suggested as an important process in cisplatin-induced nephrotoxicity [18, 19], although there is some controversy [20, 21]. In this study, we showed that the amount of Mac-2-positive macrophages was markedly increased after cisplatin treatment and Ac-YVAD-cmk effectively suppressed Mac-2-positive macrophage infiltration. Infiltrating macrophages secrete proinflammatory cytokines and chemokines into damaged kidney tissues [17]. Among them, TNF-*α* is considered a key modulator in cisplatin-induced inflammatory responses and kidney injury. A previous study showed that pharmacological and genetic inhibition of TNF-*α* attenuated cisplatin-induced increases in the expression of other proinflammatory cytokines and chemokines, resulting in the amelioration of cisplatin-induced nephrotoxicity [22]. In this study, we observed a significant increase in the expression of TNF-*α*, IL-6, MCP-1, and CXCL1 in the kidneys after cisplatin treatment. These changes were significantly attenuated by Ac-YVAD-cmk. Collectively, these results suggest that caspase-1 inhibition by Ac-YVAD-cmk effectively suppressed cisplatin-induced inflammatory responses, resulting in amelioration of kidney injury.

In conclusion, our data demonstrate that caspase-1 inhibition by Ac-YVAD-cmk protects against cisplatin-induced nephrotoxicity through inhibition of renal tubular cell apoptotic death, oxidative stress, and inflammatory responses. These results suggest that caspase-1 may be a promising pharmacological target for the prevention of cisplatin-induced kidney injury.

## Figures and Tables

**Table 1:** Primers used for quantitative real-time RT-PCR.

Gene	Primer sequence (5′ → 3′)	Product size (bp)	Annealing temperature (°C)	Reference
CYP2E1	Forward: GCATCCAAAGAGAGGCACACT	57	60	[23]
	Reverse: GGCTGGCCTTTGGTCTTTTT			
SOD2	Forward: GCTGCACCACAGCAAGCA	53	60	[17]
	Reverse: TCGGTGGCGTTGAGATTGT			
Catalase	Forward: CAAGTACAACGCTGAGAAGCCTAAG	74	60	[23]
	Reverse: CCCTTCGCAGCCATGTG			
Glutathione synthetase	Forward: TGCGGTGGTGCTACTGATTG	59	60	[17]
	Reverse: ACGGCACGCTGGTCAAA			
TNF-*α*	Forward: GACGTGGAACTGGCAGAAGAG	62	60	[24]
	Reverse: CCGCCTGGAGTTCTGGAA			
IL-6	Forward: CCAGAGATACAAAGAAATGATGG	87	60	[25]
	Reverse: ACTCCAGAAGACCAGAGGAAAT			
MCP-1	Forward: TAAAAACCTGGATCGGAACCAA	119	60	[26]
	Reverse: GCATTAGCTTCAGATTTACGGGT			
CXCL1	Forward: GGCGCCTATCGCCAATG	72	60	[27]
	Reverse: CTGGATGTTCTTGAGGTGAATCC			
GAPDH	Forward: ACTCCACTCACGGCAAATTC	170	60	[28]
	Reverse: TCTCCATGGTGGTGAAGACA			

## Data Availability

The data used to support the findings of this study are available from the corresponding author upon request.
